# A Qualitative Health Systems Effectiveness Analysis of the Prevention of Malaria in Pregnancy with Intermittent Preventive Treatment and Insecticide Treated Nets in Mali

**DOI:** 10.1371/journal.pone.0065437

**Published:** 2013-07-03

**Authors:** Jayne Webster, Kassoum Kayentao, Samba Diarra, Sory I. Diawara, Alhassane Ag Haiballa, Ogobara K. Doumbo, Jenny Hill

**Affiliations:** 1 Disease Control Department, London School of Hygiene and Tropical Medicine, London, United Kingdom; 2 Malaria Research and Training Centre, University of Bamako, Bamako, Mali; 3 Programme National de Lutte Contre le Paludisme, Bamako, Mali; 4 Child and Reproductive Health, Liverpool School of Tropical Medicine, Liverpool, United Kingdom; Tulane University School of Public Health and Tropical Medicine, United States of America

## Abstract

**Introduction:**

Delivery of intermittent preventive treatment with sulphadoxine-pyrimethamine to pregnant women (IPTp-SP) through antenatal clinic (ANC) in Mali is low, and whilst ANC delivery of insecticide treated nets (ITNs) is higher, coverage is still below national and international targets. The aim of this study was to explain quantitative data from a related study which identified ineffective processes in the delivery of these interventions in one district in Mali.

**Methods:**

In-depth interviews were conducted with health workers at the national, regional, district and health facility levels on their perceptions of reasons for the ineffective processes identified in the quantitative study, and their reported practices. Themes were coded for each ineffective process, and within these a health systems lens was used. Content analysis was used for emergent themes within this framework. MindMaps were used to display the findings.

**Results:**

Intervention specific factors for the ineffective delivery of IPTp-SP included misunderstanding of the upper limit of the gestational age at which SP could be given and side effects of SP. Incorrect practices had been recommended in training and supervision of health workers. Pregnant women who were ill on attendance at ANC were not consistently managed across health facilities. The most common reason for not offering women an ITN on their first ANC visit was if they were from outside the health facility catchment area. Broader health systems issues influencing the effectiveness of delivery of each of these interventions were also identified.

**Conclusion:**

In this setting, intervention-specific factors resulted in the ineffective delivery of IPTp-SP. These relate to complex policy guidelines, lack of guidance on how to implement the guidelines, and the institutionalising of practices that undermine the national guidelines. Interventions may be implemented and show real gains in the shorter-term whilst waiting for broader health systems issues to be addressed.

## Introduction

Intermittent preventive treatment with sulphadoxine-pyrimethamine (IPTp-SP) for pregnant women and insecticide treated nets (ITNs) are the current recommended preventive approaches in the fight against malaria in pregnancy [1]. Coverage of both interventions is well below the current targets of 100% for IPTp-SP and ITNs [2].

The delivery channel for IPTp-SP is almost exclusively antenatal clinics (ANC). Although data from the demographic and health surveys (DHS) would suggest that a varying proportion of pregnant women across countries of sub-Saharan Africa (SSA) obtain SP for prevention during pregnancy outside of ANC, almost nothing is known about the dosages received, and from where they are received, and whether these may constitute IPTp-SP. Women should receive IPTp-SP twice during pregnancy with the first dose in the 4^th^ month of gestation and each dose at least one month apart [3]. According to the Malian national policy, IPT-SP should be given twice during pregnancy, free of charge, and should not be given to women in the ninth month of pregnancy [4]. ITNs are delivered through a multitude of channels [5], [6], with perhaps the largest proportion of ITNs currently in households in SSA having been delivered through mass campaigns [6]. Nevertheless, countries of SSA also have a policy of delivery of ITNs through ANC to pregnant women [7], and some countries have achieved considerable coverage within households through this channel [8].

In Mali both IPTp-SP and ITNs are delivered through ANC and whilst 71% of women access ANC at least once, the disparities in this access across socio-economic groups are very high [9]. The proportion of pregnant women receiving at least one dose of IPTp-SP as measured in this same DHS was just 6.0%. Given the proven continuing effectiveness of IPTp-SP in Mali [10], it is important to understand the reason for these substantial missed opportunities, such that interventions can be designed and implemented to increase the proportion of women receiving this intervention.

A study was conducted in Segou District to 1) describe the health systems algorithm for delivery of IPTp-SP and ITNs, 2) to quantify the effectiveness of each of the intermediate processes in this algorithm, and 3) to identify predictors of effectiveness of intermediate process that were found to be ineffective (Webster et al Unpublished). The health systems algorithm defines each of the processes that need to occur for pregnant women to be given effective IPTp-SP, with each of these defined as intermediate processes. Ineffective intermediate processes were defined as those that were undergone by less than 80% of pregnant women. The delivery of IPTp-SP was found to be ineffective in the study setting. Two intermediate processes were found to be particularly ineffective which were: a pregnant woman being given any SP during the ANC visit observed; and being given IPTp-SP by directly observed therapy (DOT). Methods and results of the quantitative study are summarised in Box S1.

Here we report on a qualitative study undertaken approximately 18 months after the quantitative survey that sought to understand the reasons why these intermediate processes were found to be ineffective, together with the reasons why some pregnant woman were not offered an ITN, from the perspective of the health workers in the study sites. We use a health systems lens through which to view health worker perspectives of the ineffective processes in the delivery of both IPTp and ITNs.

## Methods

### Ethics

The study was approved by the ethics committees of the Faculty of Medicine, Pharmacy, and Odonto-stomatology, University of Bamako, the London School of Hygiene and Tropical Medicine, and the Liverpool School of Tropical Medicine. Health workers gave signed consent for the in-depth interviews and for the use of anonymous quotes.

The study was undertaken in Segou District, Segou Region, Mali. The district has a total of 29 functioning health structures comprising 1 hospital, 1 district level health facility (*Centre de santé de reference*), and 27 community health centres (CSComs). At the time of the study there were 8 non-functional CSComs. The hospital serves as the regional referral centre and the *Centre de Santé de Reference* (CSRef) for district level referrals. An overview of the study setting was presented in the companion paper Webster et al Unpublished and further details of the health systems context are presented below.

### Sampling and study procedures

Health workers from the national, regional, district and health facility levels were purposively selected for the study to represent the views of a range of levels and roles within the health system. Health facilities included in the study were amongst those selected for the companion quantitative study. Health facility interviewees were either managers of the facility or ANC, or were at the frontline of delivery of ANC and delivery. Interviews were conducted with the health workers in French and the local language (Bambara), by one of the authors (SaD). Interview themes included: pregnant women's access to ANC, the services provided within ANC and the structure of these services, and the examinations and interventions delivered through ANC, including the delivery of IPTp-SP, ITNs and malaria case management and specifically health workers' perceptions of factors influencing delivery of these interventions within ANC. Interview guides were unstructured and all questions were open ended. The interviews were flexible, allowing exploration of emergent issues that were not in the original topic guide but were raised by the interviewees. Themes were thus developed inductively throughout the study, with emergent themes in one interview being included in subsequent interviews.

### Coding and analyses

The interviews were transcribed, translated and entered into NVivo version 8 for data management and analysis. All translations were verified by one of the authors (KK). After familiarisation with the data, a framework approach [11] was taken to the analysis at two levels. The first level was coding of data around the primary framework themes of: 1) health systems context; 2) being given any SP during a visit to ANC; 3) being given IPTp-SP by DOT in ANC; and 4) being offered an ITN. The secondary framework themes, were those of the six building blocks of the health system which are: governance; financing; human resources; health information; products and technologies; and service delivery [12]. Responses of interviewees within each of the four primary framework themes listed above were coded for the six building blocks. Interviewees were not directly asked about these secondary framework themes. Content analysis was used within each of the health systems building blocks to identify emergent issues and to further divide themes into emergent sub-themes where indicated by the data.

Mind maps were developed using Mindjet MindManager 2012 to provide a visible representation of the emergent issues within building blocks of the health system for each of the primary framework themes. All interviewees were assigned anonymous health worker numbers, so that neither roles, levels within the healthcare system, nor health facilities identified in order to preserve anonymity. Quotes supporting emergent themes within the primary and secondary frameworks were tabulated and used to support text on the qualitative findings and to triangulate and explain the quantitative findings in the companion paper. Issues identified in the analysis of the qualitative data were compared with respect to predictors of each of the 3 ineffective processes identified in the quantitative data as presented in the companion paper (Webster et al Unpublished).

Methods for the quantitative study are presented in the companion paper. No new analyses of quantitative data were conducted for this paper, but findings presented previously are used to strengthen, compare and contrast with the qualitative findings presented here.

## Results

A total of 18 in-depth interviews with health workers were undertaken, including 1 from the national level, 2 regional level, 5 from district level management or referral health facilities, and 10 from community level health facilities (3 facility heads/deputy heads, and 7 ANC staff). Interviewees were representatives of the malaria control programme and of the reproductive health programme, health facility heads, or staff of ANC. They had been in their respective roles for 1 to 25 years. The narrative for each of the 3 ineffective processes: receive any SP during ANC, receive IPTp-SP by DOT, and offered an ITN, as presented below are therefore based upon the perceptions of the interviewees from discussions during the in-depth interviews and includes their reported practices.

### Context

The characteristics of ANC services in the study facilities and their distance from the CSRef (Segou town centre) were presented in the companion quantitative paper (Webster et al Unpublished). CSCom had ANC twice per week, and conducted outreach via community health workers known as *relais*. Supervision from the Programme National de Lutte Contre le Paludisme (PNLP) together with the regional level to approximately 4 districts per region and 2 CSCom per district was conducted on a 6 monthly basis. It was the responsibility of the malaria and reproductive health focal points based in the CSRef to supervise the CSComs on a more frequent basis. The major limitation to the frequency of supervision was reported as resources for transport. According to the perceptions of those interviewed there were problems in communication between programmes and between levels within programmes. There was no communication between the malaria and reproductive health focal points at the regional level. The regional malaria focal point linked with the district level malaria focal point but the regional reproductive health focal point did not. There were weak links between the national level and Regional reproductive health focal points.

Salaries of health workers were reported as being paid from a variety of sources: the government, community health centre board (ASACO), mayor, and *Pays Pauvres Tres Endettes* (PPTE) funds. Those health workers paid by the government were generally the more senior staff such as medical doctors. The community financed CSComs needed to charge money from patients in order to finance the services they offered. Responses from interviewees on the generation of money for financing the facility from ANC included a number of costs to the pregnant women: an ANC ‘ticket’ on entering the facility, ANC card, ANC consultation, lab tests, gloves, prescription order and SP. The number of these cost categories that women needed to pay varied across CSComs, as did the amount that they paid for each. Fees were higher for those attending the CSCom from outside the defined catchment area of the health facility.

Planning for drug supplies was reported as being on an annual basis; amounts were requested of the Programme National de Lutte contre le Paludisme (PNLP) by regions and these were considered in the light of recommendations from a quantification committee. The orders were then placed by the Pharmacy Populaire du Mali (PPM). Once the drugs were received by the PPM and PNLP were informed, it was then the responsibility of the PNLP to distribute them to the regions. The districts and CSComs in turn, were then responsible for collecting their supplies from the region.

### Receive any SP during the observed ANC visit amongst those of eligible gestation

**Figure 1:**
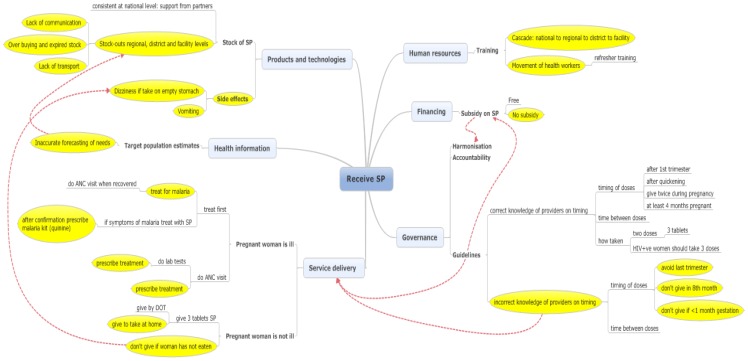
Factors reducing the effectiveness of receiving any IPTp-SP by pregnant women of eligible gestation during an ANC visit. Note: SP, sulfadoxine-pyrimethamine; ANC, antenatal clinic; DOT, directly observed treatment; HIV+ve, human immune deficiency virus positive.

Approximately two thirds of pregnant women (63.9%) who were of eligible gestation when they attended the CSRef for their first ANC visit were given SP, whilst three quarters of eligible pregnant women (74.0%; 95% CI 62.0, 83.3) were given SP on their 1^st^ visit to the CSCom (Webster et al Unpublished). Interviewees' responses on the reasons that pregnant women of eligible gestation may not receive IPTp-SP during an ANC visit included issues related to all six health systems building blocks (Figure 1). Each building block was considered in turn and links between the blocks highlighted in the text below and in supporting quotes presented in Table 1. Other relevant quotes which were not necessarily about factors influencing ineffective delivery were incorporated into the text below.

**Table 1:** Diagnosis categories for ineffective implementation of the delivery of IPTp-SP and ITNs based upon quotes of health workers.

Receive any SP
Governance: misinterpretation/lack of knowledge of national guidelines
**a) Upper limit**
*“We give [IPTp-SP] to pregnant women from 4 months of pregnancy until 7 months. At the 8^th^ month, it cannot be given.”* (Health worker 9)
*“It is the first trimester and from 8^th^ month that pregnant women must not take SP otherwise they can take it.”* (Health worker 13)
*“If they come at 7 months, their next appointment will be 8 month, we cannot give SP, we can still assess the pregnancy.”* (Health worker 1)
**a) Lower limit**
*“We provide iron tablets and SP. But if the pregnancy does not reach 1 month we do not provide SP.”* (Health worker 6)
**Governance & Financing: charging for ‘free’ SP**
*“There was SP for sale each time the free SP finished. This was in controversy to what the policy said.* “(Health worker 4)”
**Governance & Financing: lack of harmonisation**
*“For the SP, we have encountered difficulties because there are partners who started giving free SP to pregnant women while at the same time, SP was available for sale in the system. There was no harmonization”* (Health worker 10)
**Financing**
*“SP is not for sale. However, the country is so big that there are maybe some people who do don’t want to understand. Otherwise, there are directives, circulars, letters sent to all levels about the provision of free SP. Now the PPM* [National Pharmacy of Mali] *does not provide SP for sale. If there still exist SP to sell, that means that people decide themselves, to do it.”* (Health worker 10)
*“But what happen is that because of the free distribution of SP as recommended by the national level, if free SP [from the government] is not available, some health centres buy SP in another place and cannot provide it free of charge because they paid it from other sources. So there will be a problem for that“* (Health worker 4)
*“If free SP is in stock out, we have SP from other sources for sale. This is prescribed to pregnant women to buy. “* (Health worker 5)
**Human resources: cascade training**
*“The initial training is organized at the* national level *with the trainers. We train people from the region that also train trainers of district level on IPTp-SP. The districts also train the matron and the midwives.”* (Health worker 10)
**Products & technologies: SP side effects**
*“The problem is about adverse event that it creates when taken while stomach is empty. It* [SP] *can lead to dizziness, and trembles. This makes us worry about problem that could arrive like unconscious....... Some women have a stomach problem.”* (Health worker 12)
*“A lady has told me that she vomited after she took SP while she had an empty stomach. Another told me that, when she took it she was not able to do anything, even to work.”* (Health worker 14)
**Products & Technologies: stock-outs**
*“There is also a problem with the distribution, because there are some regions which even don’t need inputs and they received systematically a big quantity.”* (Health worker 4)
*“The districts started to experience stock out problem with SP. The districts have asked about SP, and we also asked the national level. Maybe the national level also does not have it...... As I said, we have sent our order, but we did not receive any answer yet...... It was about four months, we have faxed to the NMCP and the PPM, but we did not have answer yet.”* (Health worker 4)
**Service delivery: give to take at home**
*“In rural areas it is not easy because most of the women come while stomach is empty and it is not easy to take* [SP] *right away. That is why the tablets are attached together in the plastic bag so that they can take it at home after they eat.”* (Health worker 9)
*“Because we had this same problem with iron dose; we told them to take it with a piece of bread or at night when they go to bed.* (Health worker 4)
**Service delivery: don’t give to take at home**
*“We ask them to eat, otherwise we do not give them [SP] to bring at home because some don't take it. .....We often demand them to take after eating, as generally it is the market days there is selling food in the market; since some women throw them we don't give them to bring at home.”* (Health worker 1)
*“It seems that a woman delivered last Saturday and there was SP found in her bag. When we asked her why she did not take the SP she said that she forgot.......* It is good that they don’t carry at home otherwise they will not take it.” (Health worker 8)
**Service delivery: deal with illness first**
“*We start with illness by prescribing and do normal ANC later...... In case of disease, we give prescription. This does not stop the normal procedure of ANC visit.”* (Health worker 1)
*“There are two possible cases. If they come for ANC visit and we discover that they are sick, we first do the treatment and give another appointment for the ANC visit. If they come for disease and we discover that they are pregnant, we also treat the disease and ask her to come back for ANC visit.”* (Health worker 6)
**Service delivery: has malaria**
*“Some do not come for ANC visit, but only when they want malaria treatment. If we found that she suffers from malaria, we give treatment for malaria; and if she recovers we perform the normal ANC visit. We always treat the disease first before the ANC visit.”* (Health worker 8)
*“Except she has malaria; In that case we cannot give SP, because she is already having malaria treatment. We are no more in prevention but treatment. We give SP during the next ANC visit.”* (Health worker 14)

Take SP by DOT
**Human resources and products & technologies : training and side effects of SP**
*“The recommendation is to provide SP as a DOT, but there is a small problem that we are solving with midwives during the training. There are people who say that they cannot take it* [SP], *because it causes dizziness with an empty stomach. Hence the midwife asks them* [pregnant women] *to take it at home. But we provide advice to women to eat before they come to ANC so that they will take it as a DOT. Now it starts to be OK.* “(Health worker 10)
**Governance: supervision incorrect guidance**
*“We give three tablets of SP we put in a plastic bag to take at home after a meal. All the tablets should be taken together. We used to have the DOT approach before, but we changed to that one* [giving to take at home] *after a supervision which asked to give to take at home after eating. When taken without eating, it causes shakes, and can cause dizziness. That is why we don’t give it as DOT...... They* [supervisors] *said that it is not a good solution to go and find a food to eat and come back again for* (Health worker 6)
**Service delivery: give SP to take outside with food**
*“Sometimes we give them and we ask them to eat. Usually it is the market day they can find food everywhere in the market.”* (Health worker 15)
*“We ask them to buy some food outside and to take the SP, because we are not sure that they will take it at home.”* (Health worker 14)
**Service delivery: give SP to take at home with food**
*“As some women say that they did not eat, and as we know that it can create bad fatigue when you take it hungry. It can also give vomiting to some women. We then ask to take it at home after they eat.............”* (Health worker 14)
*“In our AN visit rooms, there is SP and water; we ask them to take it as DOT, but some don’t want to take it because they did not eat before and that it may give vomiting to them. We then ask them to take at home after they eat.”* (Health worker 13)
**Offer an ITN**
**Financing and Products & technologies**
*“We are provided by CSREF according to the size of our population. The CSREF gives us what we ask for. Sometimes we go there and take, and sometimes they bring it here and we pay their gas for the car that transports it.”* (Health worker 15)
*“For ITNs, there are sometimes difficulties to provide to the districts because of their transportation, also from the district to the CSCOMs. This is a big problem because, the national level was supposed to provide to the regional level, and from there to the districts and from there to the CSCOMs. There was no budget to support that, and hence there was stock out problem.”* (Health worker 10)
**Service delivery: rationing**
*“The ITNs are given only to the woman that lives in the areas covered by the CSCOM. Those coming from outside the CSCOM area do not benefit ITN. I am sorry for that fact because it is not normal that pregnant wome do not have ITN during AN visit. Before, we give ITNs to all people in and outside the CSCOM areas, but since there is insufficient ITNs, the decision has been taken to provide ITNs only to those from the CSCOM area.”* (Health worker 9)

#### Governance

According to the national guidelines eligibility for IPTp-SP amongst pregnant women attending ANC was dependent upon the gestation of their pregnancy and should be given between 4 months and 8 months gestation, inclusive; IPTp-SP should not be given before 4 months or after 8 months [4]. The reported practice from health workers in relation to gestation and giving IPTp-SP varied. Whilst some health workers interpreted the guidelines correctly, others showed either misunderstanding of, or lack of knowledge of these guidelines. The belief that IPTp-SP could not be given in the 8^th^ month was a common perception showing a misinterpretation of the upper limit of the policy. Where a woman's second ANC visit was anticipated to occur in an ineligible month of gestation, that is month 9, in some cases the first dose would be denied even though the first dose could be given in an eligible month of gestation. In other cases health workers reported that they would deny IPTp-SP to a pregnant woman of less than one month gestation, rather than 1^st^ trimester as per the guidelines.

In addition to the months of gestation guideline for when to give IPTp-SP, ‘quickening’, or movement of the baby should have occurred before the first dose is given. No misinterpretation of this guideline was found in the interviews. However, if quickening was expected by the health worker but not reported by the pregnant woman, the fundal height was measured.

Only one health worker showed any confusion on advising that 3 tablets per dose of SP should be taken, this health worker was directly involved in delivery of ANC services. No health worker interviewed reported anything other than that two doses of IPTp-SP should be taken (except in HIV positive women who, as per guidelines should get 3 doses). Reports of information given to pregnant women on the one month gap required between doses of IPTp-SP were all correct. There was a general worry that the interval between doses was often more than one month, but according to reported practice this did not prevent health workers from delivering the delayed dose.

*“I have heard that women with less weight should take 2 tablets and later on one tablet.”* (Health worker 13)

*“SP is given with at least one month interval. Sometimes it is not exactly what we do. If the women come during the 5^th^ month and she receives the dose of SP we give an appointment for the 6^th^ month. Many women don't respect that. In that case she comes during the 7^th^ month to receive the second dose.”* (Health worker 9).

According to the national guidelines IPTp-SP should be delivered to pregnant women within the health system, free of charge. However, ‘free SP’ was available only when supplied by UNICEF, and when the UNICEF SP was out of stock, SP was available for sale. Perceptions of health workers on this varied with some worried about not complying with the guidelines and others more worried about harmonisation of financing strategies.

#### Financing

Linking to guidelines and harmonisation issues above it was recognised that pregnant women should no longer be charged for SP but that charging was a consequence of stock-outs of the government provided SP. Buying SP from other sources therefore represents a stock-out coping strategy. When free SP is out of stock at the CSComs, they obtain SP from the CSRef to sell.

#### Human resources

Human resource issues specific to delivery of IPTp-SP and ITNs were not mentioned by the interviewees with the exception of training which was described as cascade training from the national to regional to district to facility. The movement of health workers between health facilities where they had been trained to other health facilities was also viewed as a problem.

#### Products and technologies

SP was generally seen as a drug for prevention of malaria, with good understanding amongst some health workers of its health impact for both the mother and baby. SP was seen by some however, as a drug which could not fully prevent malaria, but reduced the severity of disease once infected.

*“SP prevents baby and the mother against malaria, because malaria during pregnancy is very severe. SP prevent against still birth, prematurity, haemorrhages.* (Health worker 12)

*“I think it is a preventive measure; it cannot prevent them to get disease, but a woman who respect that even if she gets disease it would not be serious.”* (Health worker 7).

Factors influencing the delivery of IPTp-SP relating to products and technologies as discussed by the interviewees were twofold relating to side effects of SP and to its product and supply chain management. Side effects of SP as reported by the health workers were mainly dizziness and vomiting. Dizziness was linked to not having eaten before taking the tablets and therefore having an empty stomach. Pregnant women taking SP on an empty stomach was reported as a problem by the majority of health workers interviewed.

Interviewees variously reported that in the health facilities stock-outs were common and for periods of as long as two months, in other facilities staff reported that they had not experienced stock-outs of SP. Differing responses on stock outs of SP may be due to the interpretation of free and non-free SP, that is, the purchasing of stock from other sources by facilities when government stocks run out. The stock provided from partners was perceived as having stabilised stock at the national level.

*“The stability about SP stock out is good from 2 years to now because there is enough SP from UNICEF, USAID”* (Health worker 10).

However, problems in Product and Supply Chain Management (PSCM) at the regional, district and health facilities were described. SP stock was determined based upon population estimates but was described as inadequately taking into account malaria transmission which is variable across regions and districts of Mali. This translates to poor forecasting of needs. Based upon the interviews communication between the regions and the national level on supply can be problematic. When there was stock out at the national level, communication with timelines and expectations was sub-optimal. Supply of SP from the regions to the district and on to the health facilities was mentioned in the context of the wider PSCM systems, rather than specifically in relation to SP. The main problem was that of financing in terms of the availability of money for fuel for transport of drugs.

#### Service delivery

According to the health workers interviewed, the process through which a pregnant woman progressed on attending ANC varied depending upon whether she attended for a routine visit only, or because she was ill too. Based upon the quantitative findings 18 months before these interviews, a high proportion of women attend for routine ANC and to report an illness (27.4% CSRef and 19.0% CSComs). Where women attend for routine ANC only, the overriding reason why health workers reported that IPTp-SP is not given is because women had not eaten before attending ANC. As mentioned above there was a perception amongst the majority of health workers interviewed that SP causes dizziness and other mild symptoms when given to a pregnant woman on an empty stomach. Health workers reported two alternative courses of action where this was the case. The first was to give the SP tablets to the woman to take at home, and the second was to not give SP. Some health workers reported that the pregnant women were asked to eat and return for the dose of IPTp-SP. Where the tablets were given to take at home instructions on how to take in relation to food were reported to be given, and the adoption of special packaging to facilitate understanding of how to take was undertaken by some. Where women were not given tablets to take at home, examples of the reasons for adopting this strategy based upon experience were given and are presented in Table 1.

Based on the interviews with health workers, in most health facilities pregnant women who attend ANC with an illness are treated for the illness before performing routine ANC visits, this may therefore influence whether a pregnant woman of eligible gestation receives IPTp-SP on attendance at ANC. This is not the case in all health facilities but responses may depend upon the specific illness or disease, its severity, and the point at which during the ANC visit it is reported or identified. Several health workers reported illness as a factor promoting attendance for routine ANC and having malaria treatment as a reason for not giving IPTp-SP.

### Take SP by DOT

Where IPTp-SP is given, according to national guidelines it should be given by DOT. Amongst pregnant women of eligible gestation attending the CSRef 0% were given IPTp-SP by DOT on their first visit to ANC and 2.1% on their second visit. Amongst pregnant women attending CSComs for their first ANC and second ANC visits 24.5% and 25.4% were given IPTp-SP by DOT, respectively (Webster et al Unpublished).

**Figure 2:**
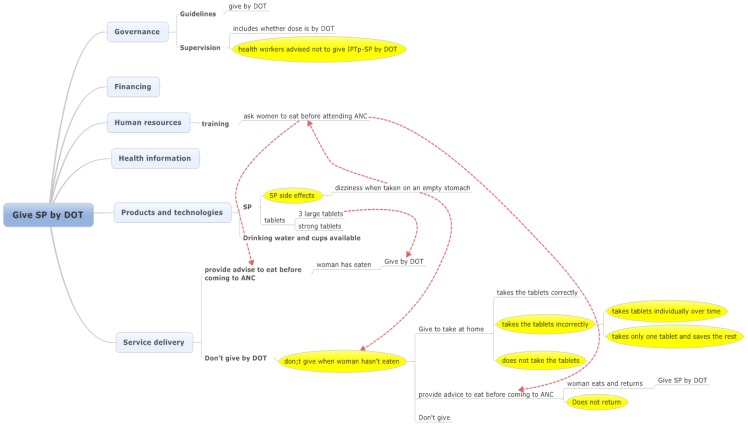
Factors reducing the effectiveness for pregnant women of receiving any IPTp-SP by DOT when of eligible gestation during an ANC visit. Note: SP, sulfadoxine-pyrimethamine; ANC, antenatal clinic; DOT, directly observed treatment; IPTp, intermittent preventive treatment.

The reasons for not giving by DOT were often the reasons that IPTp-SP was not given at all and related to the dizziness when taken by a woman who had not eaten. The categories of health systems issues impacting upon delivery of IPTp-SP by DOT are intricately linked to service delivery (Figure 2) and much less diverse than those impacting upon whether a woman receives IPTp-SP; they are therefore described together here.

Although there was acknowledgment of guidelines stating that IPTp-SP should be given by DOT, reasons that these were not practiced mostly related to side effects of IPTp-SP. It was reported that the problem of side effects of SP for pregnant women when taken on an empty stomach was included in training. During training health workers were recommended that women should be advised to eat before coming to ANC.

The giving of IPTp-SP was reported to be included in the supervisory checklists. However, supervisory visits had discouraged the use of DOT and recommended that women be given SP to take at home. This had resulted in the shift from the recommended national strategy in some facilities to one instigated by the supervisors which was out of line with national policy.

The majority of health workers interviewed had decided, through personal experience, reports of pregnant women, training, or supervision that the side effects of SP were such that it should not be taken by pregnant women who had not eaten recently. Following from this, health workers have at least three options which are to give the pregnant woman the tablets to take at home (or elsewhere) after eating, to advise the woman to eat and return for the dose by DOT, or not to give at all. Although the majority of the reports from the health workers were on their own perception of side effects of SP, the refusal to take IPTp-SP without eating was sometimes at the instigation of the pregnant women themselves.

None of the health workers interviewed said that they do not give IPTp-SP to an eligible woman, rather they said that they do not give it by DOT due to side effects and they then discussed reasons that they would not give it to be taken at home. These reasons were all down to disbelief that the woman would take the SP at all, or that she would take in incorrectly. Often these were based on the health worker having observed the tablets at a later date. Conversely, a report of side effects after taking was seen as proof that it was taken.

*“As SP should be taken as DOT, you need to sensitize women that water is here and that she must take it. We always ask if she has eaten before we give the SP............If they go home with it [SP], there is no certainty that they will take it.” (Health worker 7)*

*“Some don't take as it should be [IPTp-SP given to take at home]. Some throw it away because they don't want to take drugs or some will just keep it.” (Health worker 9)*

*“Many even may refuse to take it if they know how to take it. Some do not take for any reason. Some women throw it away because they don't like to take drugs. They also sometimes keep it somewhere. That is why we ask them to take it here.” (Health worker 15)*

*“If you give to take at home, they will not take it correctly, or they said that they take it separately the tablets with interval between the tablets.” (Health worker 12)*

*Because some say that they had a malaise after they take it. So this is the proof that they took it. (Health worker 6).*

Where IPTp-SP is given as DOT the response was often that is taken by DOT because they have water available. Reasons for giving IPTp-SP by DOT were mainly the disbelief that the women would take it otherwise.

*“Yes, they take it as a DOT because they think that the three tablets are too much for them. So that is why we give them to take in here.” (Health worker 8).*

### Offered ITN

**Figure 3:**
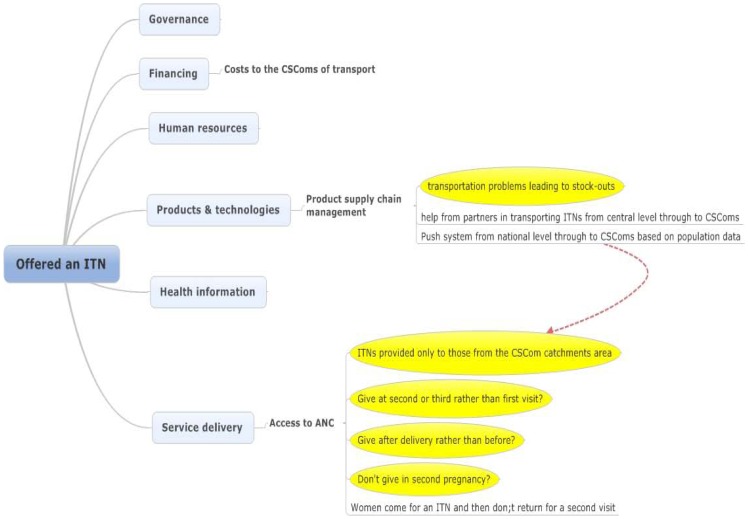
Factors reducing the effectiveness of pregnant women being offered an ITN during their first ANC visit. Note: ITN, insecticide treated net; ANC, antenatal clinic visit; CSCom, centre de santé communautaire, corresponding to community health centre.

There were a relatively limited range and number of reasons given by health workers on why women may not receive an ITN on their first visit to ANC (Figure 3). This may reflect, or be a reason for the higher proportion of eligible pregnant women receiving ITNs where they were in stock (81.7%; ITNs were stocked-out in the CSRef and in 2 CSComs) in comparison to those eligible receiving a dose of IPTp-SP (63.9% in the CSRef and 74.0% in the CSComs) or IPTp-SP by DOT (0% in the CSRef and 34.3% in the CSComs) in the quantitative study Webster et al Unpublished. Reasons given from the perception of health workers interviewed related to the supply of ITNs mainly on the transportation and cost of transportation; and to service delivery.

The number of ITNs to be supplied to the regions, districts and CSComs are based upon estimates of the catchment population at each level. This has implications for the availability of ITNs at the CSComs as discussed below, but the main problem reported by health workers in assuring a supply of ITNs for delivery to pregnant women was that of transport, and the cost of transport.

*“This scheme of provision [of ITNs] start from the central level to the DRS (regional level) is the regional level to the district, and to the CSCOM............ At each level there is a threshold and you do the order each time that threshold is reached. You don't wait until the ITNs finish to order. At central level there is scheme that provides items.” (Health worker 4)*

The perception of health workers was that much of this problem had been resolved by the support of Population Services International (PSI) who began to transport ITNs to the CSCom level.

*“PSI-MALI is responsible for this distribution from the national level to the CSCom level. So there is no stock out. “ (Health worker 4)*

*“We have PSI who supports to ensure the transport of inputs up to CSCOM level. At that time, there is no problem.............with the support from PSI the problem starts to be resolved.” (Health worker 10)*

There was one clear reason identified during the interviews for a pregnant woman on her first visit to ANC not being given an ITN, this was where a woman was from a community outside of the catchment area of the CSCom. Attendance at health facilities by those from outside the official catchment area of the health facility is common in Mali, such that the Health Management Information System (HMIS) referred to as the DESAM captures information for most indicators stratified by catchment and non-catchment population.

Three further issues discussed that may have contributed to the loss of effectiveness of delivery of ITNs, but which are dependent upon interpretation. These reasons included that the health worker would prefer to give on a second or third visit; that they felt that ITNs should be given after delivery rather than before, and that they don't give to a woman in her second pregnancy because she should have already received two ITNs, one in her first pregnancy, and the other for her child from the Expanded Programme of Immunisation (EPI) clinic. These factors all linked to the widely held perception that pregnant women attended ANC to get an ITN and then did not come again unless they were ill as reported above.

*“Women don't come as we hope because they come for ITNs. If we offer them ITNs they just disappear.......... Usually they come at ANC visit because of ITNs........”. (Health worker 8)*

*“Some women already have mosquito nets, but they are not treated. We give them, but with stock out of ITN we ask them to come during the second visit to receive their ITN. This has been changed because we give now to all women who come for the first time. There are women who try to benefit to ITNs; because they do antenatal visit in Bamako where they receive ITNs, and come here for another first visit to receive a second ITN.” (Health worker 6)*

*“They frequent other CSCOMs, if ITN are available in Famori (CSREF), they go there by paying the visit fees and get ITNs and they never come back here. That is why the coverage is higher [ANC coverage] for the first visit than the third visit.” (Health worker 7)*

*“If women receive ITN during the first visit, she will never come for subsequent visits. There was a problem of coordination. Some people think that it is good to give ITN after delivery. Some also say to wait until the third visit. Otherwise they will never come for subsequent visits once they get it during the first visit.” (Health worker 7)*

*“Those with two pregnancies must have [ITN] during their first pregnancy or during vaccination of their baby nine months after delivery................ They come to ANV more for ITNs.” (Health worker 15).*

## Discussion

This qualitative study has provided rich information on the health systems context and the delivery of IPTp-SP and ITNs within this context. Health workers discussed both their perceptions of implementation of these interventions within ANC and reported directly upon their practices. The findings explain reasons for ineffective implementation of the interventions; some of these reasons are directly related to the interventions, and others are due to the broader health systems context. The knowledge, understandings, perceptions and practices of the health workers involved are central to the health system and how it functions [13].

There are some potential limitations to this study, the first of which is that only 18 health workers were interviewed. These health workers represented a variety of cadres involved in either guidance for or direct delivery of ANC. Due to low numbers of individuals at some of these levels, there being no noteworthy differences between the perspectives of the cadres interviewed, and in order to maintain anonymity, we do not report the findings linked to level or cadre of health worker. However, the interviews undertaken produced rich information with the majority of interviewees presenting the same discussions and therefore saturation of emergent themes was reached. The coding and analysis were conducted by only one investigator but the production of the Mindmaps provided a point for confirmation of the major themes by other investigators who had conducted, transcribed and translated the interviews, and who were involved during implementation of the interviews with the inductive revision of themes on a daily basis. These therefore contributed to the reflexivity of the study [14].

There are several factors directly impacting upon the effectiveness of pregnant women receiving IPTp-SP at ANC, the first of which is misunderstanding of the policy for when the doses should be given. This misunderstanding is a link between complicated policy guidelines, poor implementation of this policy (training, information communication and leadership) and poor implementation of the intervention. There is a large literature on the impact of guidelines on implementation and factors influencing the implementability of guidelines [15], [16], [17], including reports from other settings with IPTp-SP [18]. The policy of ‘give between the 4^th^ and 8^th^ months of pregnancy’ was interpreted by many health workers as that IPTp-SP should not be given in the eighth month. This provided a plausible explanation as to why being of 4 to 6 months gestation in comparison to 7 to 8 months gestation was a quantitative predictor of receiving IPTp-SP during an ANC visit (Webster et al Unpublished). It is possible therefore to draw a direct link between health workers reporting that they don't give IPTp-SP in the 8^th^ and sometimes 7^th^ month of gestation and a reduction in the proportion of eligible women receiving this intervention.

The policy guidance that IPTp-SP should not be given during the 9^th^ month of pregnancy was probably due to safety concerns on the possibility of kernicterus in the neonate. However, IPTp-SP is now considered to have a favourable safety profile in the second and third trimesters [19]. Reduced protection due to pregnant women not receiving IPTp-SP late in the 3^rd^ trimester has been shown in Mali [10]. This was not the policy recommended by WHO but was introduced at the country level. Mali has recently dropped this recommendation in new policy guidelines [20].

The second factor influencing whether an eligible woman is given IPTp-SP is also a reason why they are not always given this intervention by DOT. This relates to side effects of IPTp-SP when taken on an empty stomach and the way in which this has been interpreted by health workers, with its cause and effect involving several building blocks of the health system. It was the perception of the majority of health workers interviewed that giving a pregnant woman IPTp-SP when she had not eaten would cause side effects. These side effects included dizziness, nausea, and fatigue most commonly. Some of these side effects were reported as experienced in their clients by the health workers. For others, the reason was that they also reported having been taught this during their training. During training some health workers had been taught that it is preferable because of the side effects (and particularly if the woman had not eaten), not to give IPTp-SP by DOT, but rather to give the pregnant woman the tablets to take at home, with instructions that they should be taken with food. Similar messages were also reported from supervision visits. This represents an agreement by the trainers that IPTp-SP should not be given on an empty stomach and a positive indication to health workers that they should not give IPTp-SP to a woman who has not eaten recently. It also provides an explanation why women of eligible gestation may not be given a dose of IPTp-SP when accessing ANC, that is, if they have not eaten before the visit. In order to get a dose of IPTp-SP they would then need to eat and return. This situation represents institutionalisation of a message which seriously undermines the national policy.

Where adverse events due to SP have been assessed in trials these have been seen in a high proportion of women 48% and 41% in Ghana [21], [22]. The side effects have included general body weakness, dizziness, vomiting, nausea, abdominal pain, diarrhoea, body itch, and body rash. However, these side effects are transient and should be managed in the ANC. Guidance on how to deal with any side effects brought about by implementing the policy should be given during training and supervision rather than advice which clearly contravenes one of the elements of the guidance, which is DOT. The IPTp-SP guidelines in Kenya for example, were updated to include the statement ‘can be given on an empty stomach’ to circumvent these problems. Without guidance, the frontline health workers had developed a number of ways of dealing with this problem, none of which were as effective in protecting pregnant women as the national policy guidance. Besides the policy guidance on what should be given, these studies have shown the need for guidance on how to give interventions. These may be termed strategy guidance, supporting, or ‘soft’ policies, but it is clear that lack of such guidance can have a profound impact on the effectiveness of implementation of interventions.

Whilst many women attend ANC whilst they are ill, there was no consistency in the reported processes through which a pregnant woman progresses if she attends ANC with symptoms of malaria or other illnesses. Some health workers reported that the illness would be dealt with first and others that routine ANC would come first and the illness would be dealt with subsequently. The range in responses may have been due to the lack of specificity of the question where respondents were thinking about different illnesses, but mostly these discussions were in the context of malaria. It is likely that the point at which the illness is reported, discussed or discovered during the visit may have an influence the process and therefore content of management. This may have been an explanation of the quantitative finding that being palpated was a positive predictor of receiving IPTp-SP in ANC, as an indicator of the woman having progressed through ANC to reach an examination in consultation. More research is needed in this area as it is possible that women who should receive IPTp-SP are not receiving it because of the way in which they progress through ANC and are diverted to deal with an illness. In Mozambique, whilst 77% of pregnant women presented in a hospital based study with symptoms of malaria, only a small proportion of them (27%) were found to be parasitaemic [23]. In a setting without diagnostic/parasitic confirmation of malaria, this would result in a large proportion of women who would should not receive treatment being treated inappropriately, and would prevent these women from being given IPTp-SP. There is relatively little information in the literature on the case management of malaria in pregnancy outside of trial settings, and we are not aware of any literature on the interplay between malaria prevention and treatment in an operational setting. The findings of this study suggest that this subject deserves more attention and more guidance needs to be given to frontline health workers on how to manage parasitaemic pregnant women attending ANC for a dose of IPTp-SP. Again strategy guidance would be key to solving ineffective implementation.

The only tangible explanation from the qualitative study as to why pregnant women would not receive an ITN on their first visit to ANC, where they were in-stock, was that of rationing to women from the defined catchment area. Other rationing decisions were made by individual health workers, such as reserving for later visits so that the woman would return, were not frequently mentioned. This supports the findings of the quantitative study that where stock of ITNs can be maintained, delivery will be effective.

Each of the issues discussed above are ones that relate directly to the specific intervention and can potentially be solved in the short term and by resolving issues in strengthened policy and strategy guidance, and communication of and training in this guidance. Whilst we recognise that having good strategy guideline documentation alone is insufficient to ensure high-quality performance of health workers [24], it is certainly a pre-requisite. Governance, supervision, and human resource issues in terms of training also need to be addressed to assure success. However, together these are relatively simple as compared with issues of financing and product and supply chain management, which both link to embedded health system problems.

Pregnant women accessing ANC were charged fees for services and medicines, often even when policy stated they were to be given free. The fees also varied between health facilities. Stock-outs of SP and ITNs linked to financing problems often due to the lack of fuel to transport supplies to the peripheral health facilities. Many of the predictors of receiving the interventions linked to fees, particularly for ITNs. The community based financing system in Mali requires the generation of finances at the health facility level to support staffing, medicines and their transport. Although prioritisation and striving for efficiency may help to some extent it is difficult to identify strategies in the immediate term to solve the financing problems. It is more important to ensure the maximum effectiveness and efficiency in the delivery of the interventions within this health system context.

Categorising reasons for loss of effectiveness across building blocks of the health system helped to gain a clear picture of not only what needs to be addressed, but also how. However, the majority of issues raised could be classified under at least two and more often more of the health systems building blocks. The concept that the health systems building blocks are not exclusive and that there are many overlaps has been well developed and discussed [12], [13]. Where this is the cases we have not necessarily presented all of these possible links, but have focussed on the block for which there is a stronger link, or for which there is a clear actionable intervention in the shorter term.

We used two methods to attempt to explain the reasons that 3 intermediate processes were ineffective as identified in the quantitative study. These methods were quantitative predictors of the effectiveness of the processes in the companion paper (Webster et al Unpublished) and qualitative analysis of in-depth interviews with health workers presented in this paper. Combining the findings from these two studies we found quantitative predictors that were explained by qualitative findings, and others for which the qualitative study provided no further explanatory information (Table 2). In addition, there were important findings from the qualitative study that were missed by the quantitative predictors. The quantitative predictors of receiving any IPTp-SP by women of eligible gestation during ANC that were explained by the qualitative study were those relating to diagnostic categories of governance: guidelines, governance: co-ordination and harmonisation, financing: subsidy and service delivery: illness. Quantitative predictors of receiving `IPTp-SP by DOT or of being offered an ITN were not explained by the qualitative findings with the exception of those relating to financing. Triangulation with the findings of the companion quantitative study strengthens the interpretation of the qualitative findings. As there was an 18 months gap between the quantitative and qualitative studies we do not suggest that the findings are directly linked in time. However, as the majority of issues raised in the qualitative study are not of a transient nature but rather are well ingrained within the structure of the facility and broader health system and within the behaviours of health workers, this time gap does not undermine the use of the qualitative study to explain the findings of the quantitative study.

**Table 2:** Factors influencing reduced effectiveness of intermediate processes in the delivery of IPTp-SP, IPTp-SP by DOT and ITNs (in the presence of required stock).

Quantitative (Adjusted predictors)	Diagnosis Category	Qualitative explanation
**Receive any SP**		
- Education (↓primary+ in CSCom)	Service delivery	No direct explanation
- Gestation(↑4 to 6 months; ref 7-8 in CSRef + CSCom)	Governance: guidelines	Misinterpretation of guidelines on gestation when IPTp-SP should be given particularly the upper limit. Includes don't give during the 8^th^ month commonly, and sometimes in the last trimester
- Reason attend ANC (↓ANC + ill in CSCom)	Service delivery: illness	Illness often dealt with first; woman may not receive routine ANC visit
- Has symptoms of malaria(↓yes in CSRef)	Service delivery: illness	Illness dealt with first; if given treatment for malaria should not be given IPTp-SP on the same visit. Note this is the correct action but will result in a decreased effectiveness measure unless such cases are removed from the effectiveness denominator
- Was palpated(↑yes in CSCom)	Service delivery	No direct explanation
- Total money spent (↑500–999; ref <500 in CSRef)	Governance:Co-ordination & harmonisationFinancing: subsidy	SP should be free and sometimes was free, but was also sometimes being sold. Those spending >CFA999 were likely to have been ill and given alternative treatment
- Spent any money in the health facility (↑yes in CSComs)	Financing:	No direct explanation
None	Products & technologies; Service delivery	Side effects of SP when a woman takes on an empty stomach. Due to side effects of SP when a woman takes on an empty stomach, IPTp-SP is given to be taken at home, or not given (possibly with the woman directed to return after eating)
**SP by DOT**		
Total money spent (↑> 500 FCFA)	Financing: subsidy	No direct explanation
None	Governance: supervision	Health workers told during supervision not to give ITPp-SP by not because of side effects but to give to take home and instruct to take with food
	Human resources: training	Health workers told during training that there are side effects if IPTp-SP given on an empty stomach and therefore not to give if the woman hasn't eaten
None	Products & technologies; Service delivery	Side effects of SP when a woman takes it on an empty stomach women therefore women not given by DOT if they have not eaten before accessing the ANC
**Offered an ITN**		
- SES (↓quintile 4)	Service delivery	No direct explanation
- woman reports malaria in consultation (↑yes)	Service delivery	No direct explanation
- was palpated (↑yes)	Service delivery	No direct explanation
- Pay for consultation (↑yes)	Financing	No direct explanation
- total money spent (↑>1,000 FCFA)	Financing	No direct explanation
None	Financing; Products & technologies	Due to rationing of ITNs, those from outside the catchment area of the CSComs are not given an ITN

Notes:

SP, Sulfadoxine-pyrimethamine; ITN, Insecticide Treated Net; CSCom, Community health center; CSRef, Reference health center; ANC, Antenatal Clinic; DOT, Direct Observed Treatment; IPTp, Intermittent Preventive Treatment; SES, Socio-economic Status; CFA, Franc XOF;

↑, arrow indicating high level; ↓, arrow indicating low level.

## Conclusions

Despite broad health systems issues being highlighted across all health systems building blocks, issues actionable in the short-term, specific to IPTp-SP and ITNs dominated. Ineffective delivery of IPTp-SP was due to misunderstanding of the upper limit of the gestational age at which it could be given, the required interval between doses and to concerns on the side effects of giving IPTp-SP to pregnant women on an empty stomach. It is likely that these factors alone account for the majority of the substantial loss in effectiveness measured in the companion quantitative study. New policy and strategy guidelines should be simplified, developed and disseminated. Other losses in effectiveness are likely due to the process of ANC for women who present with an illness. More research is required to elucidate the interaction between treatment and prevention of malaria in pregnancy at ANC.

## Supporting Information

Box S1 **Summary of companion quantitative paper.**
(DOCX)
