# Supplementary material for: A Qualitative Health Systems Effectiveness Analysis of the Prevention of Malaria in Pregnancy with Intermittent Preventive Treatment and Insecticide Treated Nets in Mali
Source: PLoS One. 2013 Jul 3;8(7):e65437. doi: 10.1371/journal.pone.0065437 (PMC3701011; doi:10.1371/journal.pone.0065437)
Supplement: Box S1 — Summary of companion quantitative paper. (DOCX) [file pone.0065437.s001.docx]

**Supporting information**

Box S1: summary of companion quantitative paper

**Prevention of malaria in pregnancy with intermittent preventive treatment and insecticide treated nets in Mali: a quantitative health systems effectiveness analysis**

**Methods**

A cross sectional study was undertaken in 10 health facilities including structured non-participant observations of the ANC process for 780 pregnant women followed by exit interviews. A health systems effectiveness algorithm defined the intermediate processes in delivery of each of the interventions. An intermediate process was classified as ineffective if <80% women successfully completed that step. Cumulative systems effectiveness represents successful coverage of women for each of the intermediate processes up to the designated point in the effectiveness algorithm. The proportion of pregnant women on their first visit and on their second visit to ANC, of eligible gestation, receiving a dose of IPTp-SP and an ITN was assessed. Predictors of each ineffective intermediate process were identified using multivariable logistic regression.

**Major findings**

Based on the national policy for IPTp-SP which includes directly observed treatment (DOT), 0% and 24.5% of pregnant women of eligible gestation on the first visit to ANC were given a dose of IPTp-SP at the district level and community level health centres respectively. Excluding the requirement for DOT systems effectiveness increased to 55.7% and 42.0% at district and community levels respectively. Two intermediate processes were ineffective which were: 'given IPTp-SP at the ANC' 63.9% and 74.0% (95% CI 62.0,83.3), and 'given IPTp-SP by DOT' 0% and 34.3% (95% CI 10.5, 69.8), at district and community levels, respectively. Delivery of ITNs was effective where they were in stock; however stock-outs were a problem, particularly at the district level. Final predictors of being given IPTp-SP at the district level facility were being 4 to 6 months gestation, reporting symptoms of malaria at ANC visit and the amount of money spent during the visit. At the community health centre facilities final predictors were being 4 to 6 months gestation, educated below primary level, attending ANC for routine visit only (not for an illness), palpated in ANC consultation, and spending any money in ANC during the visit.
